# 30 Days Wild: Development and Evaluation of a Large-Scale Nature Engagement Campaign to Improve Well-Being

**DOI:** 10.1371/journal.pone.0149777

**Published:** 2016-02-18

**Authors:** Miles Richardson, Adam Cormack, Lucy McRobert, Ralph Underhill

**Affiliations:** 1 Department of Life Sciences, College of Life and Natural Sciences, University of Derby, Derby, United Kingdom; 2 The Wildlife Trusts, Newark, United Kingdom; 3 Public Interest Research Centre, Machynlleth, Wales, United Kingdom; University of Regina, CANADA

## Abstract

There is a need to increase people’s engagement with and connection to nature, both for human well-being and the conservation of nature itself. In order to suggest ways for people to engage with nature and create a wider social context to normalise nature engagement, The Wildlife Trusts developed a mass engagement campaign, 30 Days Wild. The campaign asked people to engage with nature every day for a month. 12,400 people signed up for 30 Days Wild via an online sign-up with an estimated 18,500 taking part overall, resulting in an estimated 300,000 engagements with nature by participants. Samples of those taking part were found to have sustained increases in happiness, health, connection to nature and pro-nature behaviours. With the improvement in health being predicted by the improvement in happiness, this relationship was mediated by the change in connection to nature.

## Introduction

A number of governments are implementing policies to increase people’s engagement with and connection to nature [1,2] owing to the widespread decline of the world’s biodiversity [3] and the benefits that connection brings to pro-environmental behaviours [4] and human well-being [5]. Improving people’s connection with nature is emerging as an important societal issue (linked to well-being and biodiversity loss [6]) that has been the focus of several campaigns, for example, The Wild Network [7] and the David Suzuki 30x30 Nature Challenge (30x30.davidsuzuki.org).

However, to make a societal impact, there is a need to develop and evaluate large scale and accessible nature engagement interventions that can bring about sustained increases in people’s connectedness to nature and the associated benefits to well-being and pro-nature behaviours. Further, from a public health perspective, nature provides a new paradigm for public health [8] and is an untapped resource in upstream health promotion interventions for populations [9,10].

Given such comprehensive evidence of nature’s health benefits, health promotion partnerships with the environmental sector have been recommended [10]. Recently this has come to the fore with thirty-four conservation NGOs lobbying the UK government for one-percent of the public health budget to be invested in preventative nature-based solutions by 2018 [11]. Within this context, this paper details the development and evaluation of a mass participation campaign designed to connect people with nature and bring about the associated benefits to health, well-being and pro-nature behaviours.

The majority of people spend most of their time indoors and when outside do not consciously interact with nature [12]. As the proportion of people living in towns and cities increases, improving our engagement with nature will increasingly be based on interactions with nature within urban landscapes [13]. There is a need to consider how more ‘mundane’ or ‘nearby nature’ [14] can be valued and provide an everyday route for people to better understand their connection to nature [15] in order to improve both environmental awareness [16] and human well-being.

Previous research has argued for the need for engagement with nature [15] or has focussed on the factors that mediate human relationships with everyday nature [12]. Given the health benefits of nature, for a review see [9], research that tackles the issue of engagement with nature is needed, particularly focusing on mass-engagement that can normalise nature within people’s day-to-day lives and social contexts [12]. Simple interventions encouraging people to notice the ‘good things’ in nature each day have been shown to increase nature connection [17]. Such tools have potential value as part of wider campaigns, but there is a need to provide a variety of approaches to cater for individual differences and preferences. To this end, the *30 Days Wild* campaign set out to demonstrate the multi-faceted values of nature to individuals and to encourage people to value nature more highly in their own life, with an emphasis on commonplace and accessible nature experiences.

This paper outlines the development of the 30 Days Wild campaign before introducing the results of an evaluation of the impact of participating. In order to provide further context, the paper moves beyond simple nature exposure to consider the importance of the human-nature relationship and the links between nature engagement, well-being and pro-environmental behaviours. The evaluation sets out to test for measurable changes in happiness, general health, nature connection, and conservation behaviours.

### The Human-Nature Relationship and Connection to Nature

Technology increasingly shapes and defines us [18], to the point where people see themselves as separate from nature [19]. Technological advances have led to agricultural and then industrial revolutions that have resulted in populations adopting contrasting lives in towns and cities where they can become detached from the natural environment [20].

For the benefit of nature and the well-being of humankind, there is a need to engage people with the natural world and to encourage them to consider their relationship to it. This deeper connection with nature is fundamentally concerned with an individual’s sense of inclusion in nature, along with their sense of self [21]. Central to this is understanding our interconnectedness with nature [5] and an experiential and affective sense of belonging to the natural world [22]. The development of an ecological-self is also related to pro-environmental behaviours and a greater respect for nature [5].

Connection to nature is emerging as an important construct for well-being, alongside established societal factors such as income and education [23]. Nature connectedness is associated with pro-environmental behaviour [4] and aspects of human well-being, such as life satisfaction [22], vitality [24] and happiness [23,25]. Connection to nature has a role in human health as it helps meet the challenges of stress as well as providing resilience [24], and is considered as a relevant construct in health promotion campaigns [26].

Further, people with a greater connection to nature are more likely to spend time in green spaces [27], bringing about the more general health benefits of a simple exposure to nature outlined in a number of recent reviews [9,10,28]. These reviews cover a comprehensive and diverse body of research that demonstrates exposure to nature can lead to general health benefits such as reducing hyper-tension, respiratory tract and cardiovascular illnesses, and also to positive well-being benefits such as improved vitality and mood, and reduced anxiety. Finally, the reviews show that exposure to nature is known to restore attention capacity and mental fatigue.

### Improving Connection to Nature

Given this robust body of evidence for the positive impact of engaging with nature on human health and pro-nature behaviours, it can be argued that connection to nature is both an important goal [29] and a potential new focus for health promotion [4]. However, the applied challenge of encouraging nature connection has also been highlighted [29]. Research has shown that connection to nature can be increased in the short-term [30] with sustained increases in nature connection being achieved through specific interventions [17], but there have been few manipulations of connection to nature. Nonetheless, an understanding of the pathways and activities that can lead to increased connection to nature is emerging [17,30].

With regard to interventions, there is some evidence that educational programmes can increase nature connectedness. For example, using a measurement instrument designed for the study, two environmental education programmes (a week-long camp and multiple field trips) were found to significantly increase children’s connection to nature [31]. Similarly, two and three day workshops have led to increases in connection to nature [32], although sustained increases weren’t investigated. Similarly, education programmes with creative arts activities have led to increases in connection to nature, whereas knowledge-based activities did not improve nature connection [33]. This type of approach requires substantial time and resources with a formal engagement by those taking part. Alongside this there is a need to develop approaches that fit with people’s everyday working lives, quite often in an urban environment [15].

It is also necessary to provide appropriate routes to increase connection to nature. For example, when analysing the ‘good things’ people noticed in nature, the most common themes were the sensations of nature and noticing the sights and sounds of the natural world [17]. Growth and temporal changes were also noted often, with people also being engaged by specific aspects of nature, including the activity of wildlife, beauty and wonder, and how the weather interacts with the natural environment. This work provides a framework for connecting people with nature in order to realise the associated benefits for health and well-being.

### The 30 Days Wild Approach

As many people tend not to consciously engage with nature [12] there is a need to draw attention to the natural world [17] and suggest activities for people to engage with nature. This also creates a wider social context in order to normalise nature engagement. Research suggests that online campaigns can engage large populations and motivate people to behavioural change [34]. In the case of *30 Days Wild*, mass engagement through a clear campaign identity and a simple call to action to ‘do something wild every day’ during June 2015 provided a framework to promote engagement with nature through a wide range of suggested activities. Sharing participation via social media allowed the creation of a wider social context to encourage, extend and maintain participation. An emphasis was also placed on self-direction, in that people were encouraged to be creative and design their own activities.

The suggested activities were labelled as ‘Random Acts of Wildness’, with approximately 180 everyday activities being proposed during the campaign development phase. In order to develop a wide variety of potential activities, two categories were used, each with a three point scale:

Level of immersion: ‘full’, e.g. activities which required an intense level of dedication or time like climbing a mountain, ‘intermediary’, e.g. activities which required a medium level of involvement like identifying something new in nature, and ‘momentary’, e.g. sensory or fleeting activities like smelling a flower.

Technicality: ‘high’, e.g. requires specialist location or expert help, ‘intermediary’, e.g. activities which could require a field guide or online support, and ‘none’, e.g. activities which didn’t require any knowledge or expertise.

The activities were also divided into four main types:

Noticing–more momentary and transient experiences and activities, e.g. taking a moment to smell a wild flower or to briefly watch a butterfly.

Sharing–sharing nature experiences and activities and how they made the participant feel. The intention here was to prompt self-contemplation [30] and thinking about the value of nature. Sharing was also a way to amplify awareness of the campaign, particularly via social media.

Doing–activities that could directly or indirectly benefit nature, e.g. leaving a patch of grass to grow long, avoiding use of pesticides in the garden, contacting a local elected representative about a wildlife issue.

Connecting–activities that could help the participant to forge a stronger connection with nature. This was a broad category and examples included eating something wild (e.g. a fresh hawthorn leaf), following a bee, creating art from natural objects, and exploring a local wild place for the first time.

The activities and precise wording were then refined with reference to five pathways to nature connection currently being developed in on-going research at the University of Derby. Identified through two online surveys structured around the nine values of the biophilia hypothesis [35], these pathways include: contact, emotion, meaning, compassion and engagement with natural beauty. All of these pathways were found to be predictors of connection with nature, whereas knowledge-based activities were not. This research also found that a walking intervention, with activities operationalising the identified pathways, can significantly increase connection to nature, compared to walking in nature alone or walking in and engaging with the built environment. Therefore, where possible, reference was made to these five pathways, with purely information and knowledge-based activities kept to a minimum.

The ‘good things’ in nature were also considered during this review in order to ensure that the suggested activities tapped into aspects of nature that have been found to be valued by people when they connect with nature [17]. As an example, this process revealed that potential activities for *30 Days Wild* with an emotional pathway were scarce in number, so suggestions for revisions were made, such as changing ‘go for a lunchtime walk; what did you see?’ to ‘go for a lunchtime walk; how did it make you feel?’.

Further, as individuals and their behaviours are influenced by values and emotions, the framing of conservation communications was considered [36]. For example the activity of walking barefoot in nature was likely to engage the value ‘unity with nature’ and the activity ‘scribble a poem’ was likely to engage the value ‘creativity’. This values screening process was undertaken by The Public Interest Research Centre. Decisions were then made regarding which of the activities to include in the campaign.

This work also informed the choice of frames used in the promotion of the campaign, notably highlighting how amazing nature is, encouraging shared experiences in nature and supporting self-direction and creativity in the context of the natural world. This process culminated in the final list of 101 Random Acts of Wildness activities.

The 101 activities were then promoted on the *30 Days Wild* website to guide and inspire people to find something wild to do each day of the month. Thirty activities were also published in a campaign booklet that was sent to participants who signed up online, either in a digital or paper format. Participants were encouraged to share their ideas and experiences on blogs and social media.

The activities formed the key content for the campaign and were presented within a wider package of materials for participants, including the booklet, a wall-chart (to record activities each day during June), badges and stickers. A number of communications resources were developed, including a bloggers pack, a twitter account, a Facebook group, social media graphics, a campaign film, a series of 12 emails sent to participants throughout June and dedicated web pages at mywildlife.org.uk/30DaysWild

The *30 Days Wild* campaign was not framed as a public health or health promotion campaign, although benefits to health and well-being were a likely outcome. The primary objective was to encourage people to make more time for nature in their lives and thus value nature more highly. There was a focus on ‘everyday nature’, e.g., suggesting that people convert a walk to the bus stop into a walk with wildlife.

The evaluation focussed on the following outcomes: connection to nature; well-being; improved health; and an increase in conservation behaviours. Not only would these outcomes have an inherent societal benefit, they would also demonstrate success to a wide audience.

## Materials and Methods

### Design

The evaluation used a 1x3 (A-B-B) repeated measures time-series design where self-reported scores were taken at three time-points: pre-participation, post-participation and follow-up at two months. The clear rationale, robust theoretical basis, clear delivery content and method towards defined outcomes, meet checklist criteria for public health interventions [37]. This design approach can provide convincing evidence that an intervention is effective within a public health context [38] and has a long history of success in non-medical research [39], especially where an intervention has little potential for harm [40].

**Fig 1:**
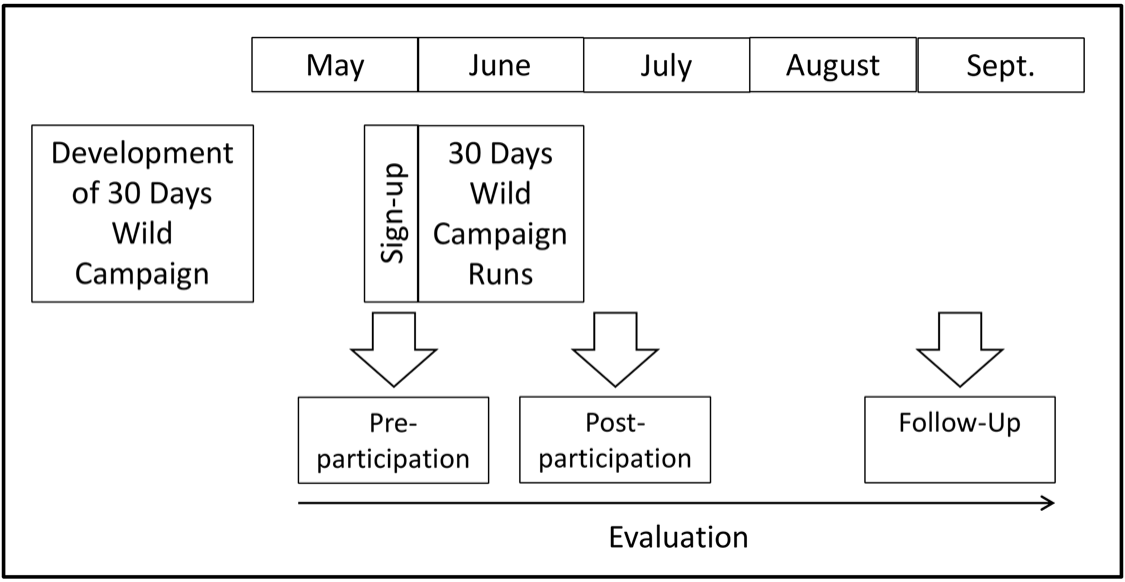
Chronology of 30 Days Wild and its Evaluation.

As with similar designs for evaluations of applied nature activities [33] and large-scale health promotion campaigns [41] a Randomised Controlled Trial (RCT) was not a practical option. Further, the chosen approach to design is acceptable when measures can be expected to be relatively stable over time [40]. For example, in the UK, monthly figures (n = 1000) from the Office of National Statistics (ONS) show that happiness remains constant through May to October with any variation in late Autumn and early Spring being of small magnitudes (e.g. approximately 1%), [42]. The chronology of the campaign development and evaluation is mapped out in Fig 1.

### Participants

Of the 12,400 people who formally signed up for *30 Days Wild*, 2305 successfully completed the baseline pre-participation survey, 344 both the pre-participation and the post-participation survey in July, 269 both the pre-participation and the follow-up survey in September, and 126 participants successfully completed all three time points (Table 1). To ensure a high level of engagement with the thirty days available, only those who had signed up for *30 Days Wild* within the first week of June were included in the post-participation surveys.

**Table 1:** Participant ages and gender by time point.

	N	Mean Age	SD	Males	Females	Age Range
Pre-participation	2203	41.07	12.37	295	1908	18 to 80
Post-participation	344	43.54	12.57	44	300	19 to 80
Follow-up	269	43.19	13.42	30	239	18 to 80
All time points	126	43.20	12.30	15	111	22 to 71

### Materials

A survey was used to evaluate the impact of the campaign on participants. The survey was framed as a ‘Wildness Quiz’ in order to engage participants within the communications style of the campaign and also to reduce demand characteristics. This survey did not need to be extensive; single item measures are routinely used by the ONS to monitor population well-being [42].

In addition to questions about age and gender, the survey measured connection to nature, pro-nature behaviour, health and well-being. Well-being was measured with a single question on general happiness with an 11-point scale that has been shown to correlate highly with multi-item well-being scales (e.g. Oxford Happiness Index and Satisfaction with Life Scale) and offers a reliable and valid measure of well-being for community surveys [43]. Health was measured with a single item based on a 5-point rating scale from excellent to poor, which has been used successfully in a range of published research, for example [44]. Nature connection was measured with single item about Inclusion of Nature in Self [45]. This measure assesses the proportion of nature included in one’s concept of self, using pairs of circles (self and nature) with a varying degree of overlap. An online version of this tool was used in which participants scored 0 to 100 depending on the degree of overlap [46]. The Inclusion of Nature in Self (INS) scale measures a cognitive aspect of connection to nature and has been found to correlate with environmental attitudes and behaviours [47] as well as aspects of well-being such as anxiety [23].

Finally, as a measure of pro-nature conservation behaviours does not exist, a series of five questions on this theme were developed. These asked about participants’ actions (such as feeding birds, growing wildlife friendly plants and doing conservation work away from home) using ‘Yes, I do this’ and ‘No, I don’t do this’ as response options. The number of ‘yes’ responses were summed. In order to gauge participation level, the post-participation surveys included a question asking participants to estimate how many days they had done something ‘wild’ in June.

### Ethics Statement

All participants provided informed consent, recorded via an online tick box labelled ‘Yes–I accept’ that followed a written brief on a ‘Your Consent’ page. The Psychology Research Ethics Committee at the University of Derby approved the study and consent procedure.

### Procedure

Invitations to complete the ‘Wildness Quiz’ were included in the sign-up process for *30 Days Wild*. Participants who followed this link were directed to a consent page prior to a page featuring the questions and a short debrief. Participants then spent time taking part in their selected activities as described above, in an unmonitored fashion. Those who had completed the pre-participation survey were invited by e-mail to complete the post-participation survey in July and the follow-up survey in September.

### Data Analysis

SPSS version 22 was used for all analyses. Paired samples t-tests were used to investigate differences between pre and post participation, and pre and follow-up results. To investigate differences between all three time points a 1 x 3 (Time) repeated measures ANOVA with pairwise comparisons was conducted. To explore the relationship between improvements in nature connection, happiness and health over June and the mechanism behind the change in health, a mediation analysis was conducted. A Bootstrapping approach was used with 5,000 bootstrap re-samples at a 95% confidence interval, as this method has more power than the sobel or causal steps tests [48]. Multiple regression analysis was also undertaken, but is not reported owing to similar results.

## Results

### Pre to Post-participation to Follow-up Results

There were statistically significant increases from pre-participation baseline to post-participation for connection to nature, health, happiness and number of reported conservation behaviours (Tables 2 and 3). There were also statistically significant increases from pre-participation baseline to follow-up for connection to nature, health, happiness and number of reported conservation behaviours (Tables 2 and 3). ANOVA analysis of pre to post-participation to follow-up results revealed that time had a statistically significant impact on connection to nature, happiness, health and conservation behaviour scores (Table 3). Pairwise comparisons indicted significant differences (*p* < 0.01) between pre-participation and post-participation, and significant differences between pre-participation and follow-up for all four outcome measures.

**Table 2:** Pre, post-participation and follow-up means and standard deviations for the four outcome measures.

	Pre-participation		Post-participation		Follow-up	
	Mean	SD	Mean	SD	Mean	SD
Connection to Nature	52.79	23.32	59.36	21.18	-	-
Conservation Behaviours	3.36	1.11	3.61	1.00	-	-
Health	3.60	0.95	3.80	0.92	-	-
Happiness	7.25	1.72	7.72	1.50	-	-
Connection to Nature	53.68	23.18	-	-	62.64	22.51
Conservation Behaviours	3.37	1.13	-	-	3.65	1.01
Health	3.66	0.95	-	-	3.86	0.98
Happiness	7.37	1.63	-	-	7.86	1.45
Connection to Nature	53.17	23.62	60.95	22.75	66.19	21.54
Conservation Behaviours	3.41	1.16	3.68	1.02	3.74	1.00
Health	3.56	1.04	3.78	1.05	3.83	1.01
Happiness	7.24	1.83	7.76	1.54	7.87	1.54

**Table 3:** Summary of paired t-tests and repeated measures ANOVA analyses.

	Pre to Post		Pre to Follow-up		1 x 3 ANOVA		
	T(343)	d	T(268)	d	F	df	η^2^
Connection to Nature	5.51	0.29	6.21	0.39	22.88	1.89, 236.79	0.16
Conservation Behaviours	6.16	0.24	5.68	0.26	16.54	1.83, 222.86	0.12
Health	5.71	0.21	5.03	0.21	10.74	2, 250	0.08
Happiness	6.65	0.29	5.60	0.32	15.29	1.78, 222.97	0.11

All significant at *p* < 0.01

### Relationship between Health, Happiness and Nature Connection

Mediation analysis on the pre to post-participation data was conducted using improvement in happiness as a predictor of improvement in health, with improvement in nature connectedness as a mediator. The model met all the criteria for mediation [49]. Both sobel and bootstrap results show the indirect effect to be significant (Table 4). The analysis was repeated on the pre-participation to follow-up data. The mediation model met all the criteria for mediation. Both sobel and bootstrap results show the indirect effect to be significant (Table 5).

**Table 4:** Simple Mediation of the Indirect Effects of Happiness and Nature Connection on Health (5000 Bootstrap Samples).

	β	SE	t	p
Happiness to Health: Total Effect	.145	.026	5.623	< 0.01
Happiness to Nature Connection	3.805	.877	4.336	< 0.01
Nature Connection to Health controlling for Happiness	.005	.002	3.156	< 0.01
Happiness to Health controlling for Nature Connection: Direct Effect	.126	.026	4.826	< 0.01
	Z	P	LL95%CI	UL95%CI
Sobel and Effect	2.509	0.012	.006	.035

**Table 5:** Simple Mediation of the Indirect Effects of Happiness and Nature Connection on Health (5000 Bootstrap Samples).

	β	SE	t	p
Happiness to Health: Total Effect	.100	.027	3.614	< 0.01
Happiness to Nature Connection	4.594	.961	4.783	< 0.01
Nature Connection to Health controlling for Happiness	.007	.002	4.181	< 0.01
Happiness to Health controlling for Nature Connection: Direct Effect	.064	.027	2.400	< 0.01
	Z	P	LL95%CI	UL95%CI
Sobel and Effect	3.110	0.019	.013	.054

## Discussion

There is a need to increase people’s engagement with and connection to nature for nature’s and human well-being. The results of the evaluation show that the mass engagement campaign, *30 Days Wild*, led to sustained increases in connection to nature, also bringing associated benefits to happiness, health, and pro-nature behaviours. In doing so it met the criteria for success of population-based public health interventions [39]. The campaign approach and evaluation results are of value as they combine, and offer potential solutions for, societal issues of well-being and nature conservation. The evaluation also extends previous research on large-scale campaigns that have focussed on specific health outcomes to engage large populations and motivate people to specific behaviour change [34]. Within this context it should be noted that the promotion of *30 Days Wild* did not focus on health, well-being or conservation outcomes, but simply conveyed a message that being in contact with nature makes life better.

The campaign success in engaging a large number of participants and delivering outcomes is notable given governmental and public health interest in policies and interventions to increase human well-being. There are also indications that the *30 Days Wild* campaign can lead to nature being valued [14], bringing about improved connection to nature and pro-conservation behaviours [13,15] within the context of everyday life. The evaluation provides evidence that conscious connections to nature can and should be part of a healthy lifestyle.

The results also provide some indication of the relationship between connection to nature, well-being and health outcomes. The replicated mediation analysis suggests that the improvement in reported general health was related to the improvement in happiness, mediated by the increase in nature connection. Links between nature connection and happiness have been established previously [50], but understanding the link to health is of value. Our findings suggest that connection to nature may provide people with resilience to meet the challenges of everyday life, while also facilitating exercise, social contact and a sense of purpose [24]. The mechanisms require more focused research, but this result highlights the role of nature in a happy and healthy lifestyle and our embedded place in the natural world [8]. This adds important dimensions to our paradigm for human well-being.

Although the design observed good practice [37–41], limitations and key assumptions should be acknowledged. Firstly, although healthy, the sample size as a proportion of those taking part was very small. The evaluation was kept short so as to encourage participation, but it is acknowledged that the results may not fully reflect the outcomes experienced by the majority of those taking part. For example, those completing all three time points may have been motivated by a greater connection to nature. Secondly, there is an assumption that those taking part did engage with nature in some way. Based on post-hoc estimates of days ‘wild’, the majority of participants reported engagement of 25 days or more, but the reliability of such an estimate can be questioned. Without observational data this is hard to test further. A third assumption is that taking part led to measureable experiences, with responses that could be detected by the measures used. Finally, participants may have been primed by the questions and therefore offered more positive outcomes in July and then again two months later in September. This is difficult to overcome and an issue with any pre-post self-report evaluation of an intervention. However, a recent systematic review found little evidence to support such demand characteristic effects [51]. Within the research identified by the review were two RCT studies of a similar design to the present research. These showed that awareness of study aims did not impact self-reported outcomes [52,53]. Further, the campaign was not widely promoted with regards to well-being, and precautions were taken to limit priming. For example participants were not aware of the purpose of the study which was presented as a ‘Wildness Quiz’.

Although single item measures were used to enhance the accessibility of the campaign, the sustained increase across all four measures gives confidence in the results. The replicated mediation analysis showing a consistent relationship between happiness, health and connection to nature also provides further confidence in the findings. Further, the single-item measures used have good validity and reliability, with a similar approach used in official surveys of population well-being [42]. Finally, health, well-being and nature connection may have some seasonal variation, but without intervention such measures are known to be relatively stable. For example, in the UK, ONS statistics [42] show that happiness remains constant May through to October with any variation in late Autumn and early Spring being of small magnitudes (e.g. approximately 1%), compared to 6% after participating in 30 Days Wild.

A repeated measures time-series design is a proven approach in evaluations of such scale and type [38], and key criteria were met in this case [37]. RCT would provide the best evidence in future research, but a sequence of evaluations of growing complexity, carried out on the basis of earlier, more straightforward work is recommended [54,38] and this should include economic significance [9]. In the current study, the sustained increases over two-months, effect size and mediation analysis give confidence an intervention effect [39] was achieved, providing a basis for more robust measures and designs. Such promising results provide motivation and justification for extended evaluations of future campaigns.

A further limitation was gender imbalance in the (self-selecting) participants, the vast majority of whom were female. There is a need for further research to establish the reasons for the gender difference and an investigation of approaches that would engage men more successfully. Although complex, the evaluation of future campaigns could endeavour to record the actual activities users participated in. Bringing the activities used during participation into the analysis would allow examination of their appeal to each gender, and also of their relationship to the outcomes. The most effective activities could then be identified and promoted.

Effectiveness returns us to the rationale. The need to address global issues like declining biodiversity and the need for healthy lifestyles that reduce demands on our health services can both benefit from increasing people’s connection to nature. Such grand challenges require large-scale upstream interventions. *30 Days Wild* provides good evidence that progress can be made through partnerships with the environmental sector that use nature as a new paradigm for well-being [10]. By asking people to make room in their lives for nature, every day, for thirty days and to share their experience through social media, interventions can produce sustained increases in connection to nature, happiness, health, and pro-nature behaviours. This suggests there is the potential to apply *30 Days Wild* to millions of people within schools, workplaces and local neighbourhoods each year, so that they and the natural world can enjoy the ensuing benefits.

## Supporting Information

S1 File Pre and Post Data. (CSV)

S2 File Pre and Follow-up Data. (CSV)

S3 File Pre, Post and Follow-up Data. (CSV)
